# Injuring Business

**Published:** 1910-07

**Authors:** 


### EDITORIAL DEPARTMENT.

### INJURING BUSINESS.

Abolish the "saloon?" Never! It will injure business! Statutory Prohibition? It is unconstitutional. We have a statute which prohibits crime; a statute which prohibits gambling (although some of our immaculate and angelic Senators were caught red-handed in the act, and Thomas, like Sisyphus, was punished for telling tales); we have a statute prohibiting racing, prize fighting, bucket shops and numerous other penal offenses; against sale of cocaine and morphine. In fact, all statutes are prohibitive. But a statute prohibiting the indiscriminate retailing of a poison that makes widows and orphans, criminals, paupers and lunatics; that causes the disruption of homes and families; the wail of heart-broken wives and the cry of hungry children, will injure business. Hands off! Don't touch the sacred *fetich*—the dreadful saloon! Better hearts break and children starve, chains clank and maniacs rave, than injure business.

I saw a cartoon recently that impressed me. I wish I had the skill displayed by Gustav Dore in depicting Dante's infernal regions. I'd copy that picture on a canvas a mile square and stretch it from the dome of the Texas Capitol to that of the Texas University.

It represented a workingman just returned to his humble little home—Saturday night, no doubt,—wife and two children, a boy and a girl. He was in the act of taking off his shoes,—bending over, with a drunken leer on his face. His little boy stood by him with his hand on papa's shoulder. His wife, some feet away, had her face buried in her hands, and her little girl stood by her in silent sorrow. I could easily imagine the heart-breaking sobs and see the convulsive shivers of her poor body as she listened, evidently, to the man's curses and abuses. His week's wages had gone to pay Charlie, the barkeeper, who had trusted him for $6.00 worth of poison during the week, and even lent him a dollar! Seven dollars out of his $12 that he had earned, he said, by nailing on shingles in the sun—six days. Wasn't it his? Hadn't he earned it? Wasn't he entitled to a little enjoyment? Where was his much-vaunted personal rights, if he couldn't spend his own money? Nothing to eat in the house. Where was the other $5.00? "I spent it on the boys. They set 'em up for me last Saturday night."

Oh Cone, oh Sterling,—don't abolish the saloon. It will injure business—Charlie's business.

My fellow sinners who read the "Red Back," do you not recall the occurrence at Dallas lately, where the baby died, pulling at the empty breast of a skeleton mother, whose husband, then before the court for beating her, had that day stolen and sold for whisky the last handful of flour in the house? Why try to reason with such brutes? Abolish the saloon? Never! Let mother and babe starve, rather than injure business or deprive these monsters of their personal rights. Fellow sinners, in how many workingmen's homes in Austin, in Dallas, San Antonio, Galveston, Fort Worth and Houston can this picture be verified any Saturday night? Ah, how many wronged wives suffer in silence—that Jack may make business for Charlies—and enjoy his personal rights? Merciful God! Is there *no* power in heaven or earth to save me and my babies—Jack's babies—from Charlie's dreadful saloon? Prayer is unavailing—Jesus can't help me; the Governor can't, the Legislature will not, the courts are powerless. Jack! oh Jack, father of my babies—my lover—so gentle so kind, so loving. Jack—so brutal, so course, so cruel—when making business for "Charlie"!

